# Factors influencing nurses self-efficacy two years after the COVID-19 outbreak: A cross-sectional study in Wuhan, China

**DOI:** 10.1097/MD.0000000000035059

**Published:** 2023-09-08

**Authors:** Wen Li, Zhiying Wan, Yunyan XianYu

**Affiliations:** a Master of Medicine, Department of Nursing, Renmin Hospital of Wuhan University, Wuhan, Hubei Province, China; b Master of Medicine, Department of Psychiatry, Renmin Hospital of Wuhan University, Wuhan, Hubei Province, China.

**Keywords:** COVID-19, mental health, nurses, resilience, self-efficacy

## Abstract

This study explored the anxiety, depression, and self-efficacy of nurses in Wuhan, China 2 years after the corona virus disease 2019 outbreak. A total of 552 nurses were enrolled in the study. Four well-established test tools were applied: The 9-item patient health questionnaire, The 7-item generalized anxiety disorder, generalized self-efficacy scale, Connor Davidson resilience scale. Twenty-eight points twenty-six percentage of the nurses had mild depression, and 5.62% had moderate or severe depression. Twenty-one points seventy-four percentage of nurses had mild anxiety and 1.82% had moderate or severe anxiety. The average score of self-efficacies is negatively correlated with the average score of the 9-item patient health questionnaire (r = −0.303, *P* < .01), and the7-item generalized anxiety disorder (r = −0.275, *P* < .01). The average score of self-efficacies is correlated with the resilience score (*R* = 0.799, *P* < .01). Through multiple linear regression analysis, the tenacity dimension and monthly income are most closely related to the sense of self-efficacy. Nurses self-efficacy and resilience are important factors in promoting their psychological well-being. This study suggests that increasing the salary and providing some strategies to increase nurses mental tenacity can promote self-efficacy.

## 1. Introduction

The corona virus disease 2019 (COVID-19) pandemic has increased the mental health problems of healthcare workers.^[1–3]^ particularly nurses, in terms of fear, increased levels of anxiety, depression, burnout, and PTSD symptoms, because of workplace violence from thirds, fear of getting infected, and high emotional demands.^[4]^COVID-19 has increased levels of anxiety and burnout already high before the pandemic.^[5]^ Wuhan was the epicenter of the initial outbreak of the COVID-19 pandemic. When COVID-19 broke out in Wuhan in 2020, general wards were quickly modified into isolation wards, and healthcare providers who did not have infectious disease expertise stepped up to provide care for patients with COVID-19.^[6]^ Various mental health problems exist among medical personnel fighting against COVID-19 in Wuhan in early 2020. A survey revealed that 40% to 64% of the medical staff suffered from certain degrees of anxiety, and depression.^[7]^ The mental health of front-line nurses in Wuhan outcomes was negatively correlated with self-efficacy, resilience, social support, and front-line work willingness.^[8]^ The prevalence of insomnia among front-line nurses in Wuhan was 52.8 to 60%.^[9,10]^ Insomnia of front-line nurses in Wuhan was related to huge stress at work and prolonged working hours, perceived negative feelings, and missing their family. Nurses took caring for patients as “this is my duty”. They showed strong resilience in their fight against the pandemic, and this quality of resilience was related to the various support from their colleagues friends, relatives, and the whole society.^[6]^

After COVID-19 was almost under control in China by April 2020,^[11–13]^ the hospitals in Wuhan were open normally, implementing strict normalized measures of COVID-19 prevention and control.^[14]^ Nurses, as the largest part of health workers or the largest part of the healthcare system, their post-epidemic mental health problems should be our great concern.^[15]^ According to research, 1 year after the outbreak, SARS survivors still had elevated stress levels and worrying levels of psychological distress. Healthcare workers showed significantly higher stress levels in 2004 and had higher depression, anxiety, and post-traumatic symptoms than those non-healthcare workers.^[16]^ Another research revealed that the incidence of new episodes of psychiatric disorders after the SARS outbreak was similar to or lower than community incidence rates, which may indicate the resilience of healthcare workers who continued to work in hospitals 1 to 2 years after the SARS outbreak.^[17]^ According to Bandura theory, self-efficacy is the mediator between knowledge and action. Resilience is the personality trait of having positive dispositions which enable individuals to cope with stressful situations.^[18]^ Self-efficacy and resilience play an important role in the individual response to challenges.^[19]^ Self-efficacy can increase 1’s confidence.^[20]^ Coping self-efficacy is found to ameliorate the effect of psychological distress on nurses traumatic experiences.^[21]^

When the COVID-19 outbreak occurred in 2020, there were numerous reports about the mental health issues faced by nurses in Wuhan. However, 2 years after the COVID-19 pandemic broke out, there is almost no reporting on the mental well-being of nurses in Wuhan, and there was no research focusing on the mental health problems, self-efficacy, and resilience of those anti-epidemic nurses in Wuhan in 2020. The nurses included in this study were all nurses who worked in the designated hospitals for the treatment of severe COVID-19 patients during the outbreak. They were directly in the epidemic center during the outbreak of COVID-19, and their mental health problems should be paid continuous attention. This study aimed to investigate the psychological state of nurses in Wuhan 2 years after the COVID-19 outbreak, and the impact of nurses self-efficacy on their psychological state, to provide a basis for public health authorities to set plans or rules to care for nurses mental health.

## 2. Materials and methods

### 2.1. Study design

A cross-sectional study was conducted among nurses in Wuhan, China from March 7 to March 15, 2022, 2 years after the outbreak of the COVID-19 pandemic. This study was approved by the Ethical Committee of the Renmin Hospital of Wuhan University (WDRY2023-K045).

### 2.2. Participants and procedures

This study took nurses from a general hospital in Wuhan, China as the respondents. A convenient sampling method was adopted. We used G*Power software to calculate the required sample size with the following assumptions: a medium effect size of 0.15, an a of 0.05, a power of 0.95, and multiple linear regression with 15 independent variables. Based on these assumptions, 199 nurses were needed. In this study, a total of 552 nurses voluntarily completed the questionnaires. It was adequate to run the analysis. The inclusion criteria were as follows: Registered nurses; Working in the hospital during the COVID-19 outbreak. Exclusion criteria: Nurses having left their jobs; Absent nurses due to maternity leave, hospitalization, and other reasons during the survey. All questionnaires required for the survey were made digitally into QR codes. We trained the surveyors to make sure their understanding of the scale was consistent with each other. The trained surveyors went to each clinical department to collect data. They would inform participants of the content and significance of this study in advance. The respondents scanned the QR code to complete the anonymous questionnaires with the principle of informed consent. The surveyors there were responsible for the explanation and details on how to complete those questionnaires. All questions were required to be answered and the questionnaires should be done only once on the spot by each single ID account. All data were committed to confidentiality.

### 2.3. Measurements

#### 2.3.1. Socio-demographic variables

The study collected demographic data from the nurses who participated in the survey, including sex, age, work department, marital status, working years, education background, professional title, current monthly income, and the length of weekly work. Additionally, they were asked about variables specific to COVID-19 (traumatized or not during the outbreak, infected or isolated as a close contact or not, and what your specific job was during the outbreak).

#### 2.3.2. The 9-item patient health questionnaire (PHQ-9)

The PHQ-9 is a self-report measure used to assess the severity of depression. It consists of 9 items and is scored on a Likert scale ranging from 0 to 3. The total scores were categorized as follows: minimal/no depression (0–4), mild depression (5–9), moderate depression (10–14), or severe depression (15–27).^[22]^ The psychometric properties of the PHQ-9 have been previously confirmed in Chinese populations, with a Cronbach alpha of 0.915.^[23]^

#### 2.3.3. The 7-item generalized anxiety disorder (GAD-7)

The GAD-7 is a self-rated scale used to evaluate the severity of anxiety and has good reliability and validity.^[24,25]^ It consists of 7 items, each scored on a 4-point scale (0–3 points). The total scores are categorized as follows: minimal/no anxiety (0–4), mild anxiety (5–9), moderate anxiety (10–14), or severe anxiety (15–21). The Cronbach alpha of the GAD-7 was 0.928.^[26]^

#### 2.3.4. Generalized self-efficacy scale

This instrument was first developed by Schwarzer and Jerusalem in 1979 and then revised in 1981. The internal consistency of the questionnaire was also estimated through Cronbach alpha, which was found to be 0.78.^[27]^ It consists of 10 items, which all measure the level of general self-efficacy. The scoring is on a 4-level Likert scale ranging from 1 to 4. An overall score between 10 and 20 is interpreted as low self-efficacy, between 21 and 30 is taken as a moderate 1, and above 30 is interpreted as high self-efficacy. Notably, the reliability and validity of the questionnaire were already established. In this study, we used the Chinese adaptation of the generalized self-efficacy scale scale.^[28]^

#### 2.3.5. Connor Davidson Resilience scale (CD-RISC)

This study used the CD-RISC, revised and translated by the Chinese scholar Yuan and his associates.^[29]^ The scale has a total of 25 items and 3 dimensions (tenacity, strength, optimism), with a score range of 0 to 4. Answering “never” scores 0 points, “rarely” scores 1 point, “sometimes” scores 2 points, “always” scores 3 points, and “almost always” scores 4 points. The points scored ranged from 0 to 100 points. Of the above 3 dimensions, tenacity was composed of questions 11 to 23, a total of 13 items, and the score range was from 0 to 52 points. The strength dimension was composed of questions 1, 5, 7, 8, 9, 10, 24, 25, a total of 8 items, and the score range was from 0 to 32 points. The optimism dimension was composed of questions 2, 3, 4, and 6, a total of 4 items, and the score range was from 0 to 16 points.^[30]^ A higher score meant better psychological resilience. Cronbach α coefficient was 0.91 in the Chinese version of the CD-RISC scale.

### 2.4. Statistical analysis

The study utilized R statistical software (version 4.0.3) for data analysis. The measurement data were described using mean ± standard deviation, while count data were described using the number of cases (percentage). The measurement data were tested through Shapiro–Wilk normal test and Levene test, and inter-group difference analysis was made through Fisher exact probability, Kruskal-Wallis H test, and Pearson chi-squared test. Spearman Rank Correlation was utilized where it did not meet the normal distribution between the 2 variables. Stepwise multiple linear regression was used and a *P* value < .05 was considered significant.

## 3. Results

### 3.1. Demographic characteristic

The average work experience of the nurses was 6.10 ± 4.65 years and their average age was 28.61 ± 4.71. Two hundred fifty-two of the nurses (45.65%) were single (1 divorced) and 300 of them (54.35%) married; 14 (2.54%) were male and 538 (97.46%) female. Twenty-eight points Twenty-six percentage of the nurses had mild depression, and 5.62% had moderate or severe depression.21.74% of nurses had mild anxiety and 1.82% had moderate or severe anxiety. The proportion of low self-efficacy, moderate self-efficacy, and high self-efficacy was 18.30%, 54.35%, and 27.35% respectively. The average score of each scale can be seen in Table 1 below. (Table 1)

**Table 1:** The mean scores of variables.

Variable	Mean ± SD
GAD-7	2.26 ± 3.05
PHQ-9	3.51 ± 3.89
GSE-10	27.73 ± 6.99
CD-RISC	64.94 ± 21.53
The strength dimension	20.95 ± 6.87
The optimism dimension	11.02 ± 3.97
The tenacity dimension	32.86 ± 11.55

CD-RISC = Connor Davidson resilience scale, GAD-7 = The 7-item generalized anxiety disorder, GSE-10 = generalized self-efficacy scale, PHQ-9 = The 9-item patient health questionnaire.

### 3.2. Relationship between demographic variables with self-efficacy

The results showed that average monthly income, COVID-19-related psychological trauma, and professional title were significant influencing factors for nurses self-efficacy (*P* < .05). (Table 2)

**Table 2:** Relationship between demographic variables with self-efficacy.

	Total (n = 552)	Self-efficacy			Fisher/c^2^/H	*P* value
		Low (n = 101)	Moderate (n = 300)	High (n = 151)		
Department						.09
Emergency department	44 (7.97)	6 (5.94)	26 (8.67)	12 (7.95)		
Buffer ward	128 (23.19)	23 (22.77)	66 (22.00)	39 (25.83)		
Internal medicine	158 (28.62)	20 (19.80)	91 (30.33)	47 (31.13)		
Surgery	44 (7.97)	6 (5.94)	25 (8.33)	13 (8.61)		
Obstetrics and gynecology	36 (6.52)	13 (12.87)	16 (5.33)	7 (4.64)		
Pediatrics	48 (8.70)	13 (12.87)	26 (8.67)	9 (5.96)		
ICU	59 (10.69)	14 (13.86)	28 (9.33)	17 (11.26)		
Mental health center	14 (2.54)	0 (0.00)	9 (3.00)	5 (3.31)		
Fever clinics	9 (1.63)	2 (1.98)	7 (2.33)	0 (0.00)		
Nucleic Acid Test	12 (2.17)	4 (3.96)	6 (2.00)	2 (1.32)		
Work experience (yr)					0.70	.70
<5	202 (36.59)	38 (37.62)	105 (35.00)	59 (39.07)		
5–10	280 (50.72)	55 (54.46)	158 (52.67)	67 (44.37)		
11–20	58 (10.51)	6 (5.94)	30 (10.00)	22 (14.57)		
>20	12 (2.17)	2 (1.98)	7 (2.33)	3 (1.99)		
Sex						.16
Male	14 (2.54)	0 (0.00)	9 (3.00)	5 (3.31)		
Female	538 (97.46)	101 (100.0)	291 (97.00)	146 (96.69)		
Age, yr					3.74	.15
<25	122 (22.10)	28 (27.72)	65 (21.67)	29 (19.21)		
25–30	301 (54.53)	54 (53.47)	166 (55.33)	81 (53.64)		
31–40	112 (20.29)	16 (15.84)	6 (20.33)	35 (23.18)		
>40	17 (3.08)	3 (2.97)	8 (2.67)	6 (3.97)		
Marital status					2.60	.27
Single/divorced	252 (45.65)	53 (52.48)	135 (45.00)	64 (42.38)		
Married	300 (54.35)	48 (47.52)	165 (55.00)	87 (57.62)		
Educational background					4.04	.13
College diploma or below	27 (4.89)	7 (6.93)	13 (4.33)	7 (4.64)		
Bachelor	508 (92.03)	94 (93.07)	276 (92.00)	138 (91.39)		
Master or higher	17 (3.08)	0 (0.00)	11 (3.67)	6 (3.97)		
Title					6.33	.04
Nurse	123 (22.28)	30 (29.70)	61 (20.33)	32 (21.19)		
Senior nurse	337 (61.05)	59 (58.42)	179 (59.67)	99 (65.56)		
Supervisor nurse	89 (16.12)	12 (11.88)	57 (19.00)	20 (13.25)		
Co-chief nurse and above	3 (0.54)	0 (0.00)	3 (1.00)	0 (0.00)		
Monthly income, CNY					21.45	<.001
≤3000	37 (6.70)	12 (11.88)	14 (4.67)	11 (7.28)		
3000–5000	240 (43.48)	57 (56.44)	130 (43.33)	53 (35.10)		
5000–10,000	233 (42.21)	32 (31.68)	127 (42.33)	74 (49.01)		
10,000–15,000	37 (6.70)	0 (0.00)	25 (8.33)	12 (7.95)		
≥15,000	5 (0.91)	0 (0.00)	4 (1.33)	1 (0.66)		
Average working hours per wk (h)					3.14	.21
<20	16 (2.90)	3 (2.97)	8 (2.67)	5 (3.31)		
20–40	268 (48.55)	48 (47.52)	139 (46.33)	81 (53.64)		
41–60	256 (46.38)	48 (47.52)	144 (48.00)	64 (42.38)		
>60	12 (2.17)	2 (1.98)	9 (3.00)	1 (0.66)		
COVID-19 psychological trauma					22.95	<.001
Yes	114 (20.65)	37 (36.63)	59 (19.67)	18 (11.92)		
No	438 (79.35)	64 (63.37)	241 (80.33)	133 (88.08)		
Coronavirus infection						.67
No	454 (82.25)	80 (79.21)	252 (84.00)	122 (80.79)		
Diagnosed	15 (2.72)	2 (1.98)	8 (2.67)	5 (3.31)		
Quarantine of close contact	83 (15.04)	19 (18.81)	40 (13.33)	24 (15.89)		
Jobs during COVID-19 outbreak in					4.915	.296
Isolation wards	437 (79.17)	77 (76.24)	243 (81.00)	117 (77.48)		
Management	65 (11.78)	17 (16.83)	28 (9.33)	20 (13.25)		
Absentees	50 (9.06)	7 (6.93)	29 (9.67)	14 (9.27)		

COVID-19 = corona virus disease 2019.

### 3.3. Correlation analysis between nurses self-efficacy and psychological resilience and mental health questionnaires

According to Spearman correlation analysis, nurses general self-efficacy was positively correlated with the psychological resilience scale and scores of all dimensions, *P* < .01, and negatively correlated with PHQ-9 score, GAD-7 score, *P < *.01. (Table 3)

**Table 3:** Correlation analysis of nurses self efficiency and psychological resilience and mental health questionnaires.

		Work experience	Age	PHQ-9	GAD-7	Strength dimension	Optimism dimension	Tenacity dimension	CD-RISC
GSE-10	*r*	0.013	0.067	−0.303	−0.275	0.762	0.678	0.802	0.799
	*P* value	.753	.117	.000	.000	.000	.000	.000	.000

CD-RISC = Connor Davidson resilience scale, GAD-7 = the7-item generalized anxiety disorder, GSE-10 = generalized self-efficacy scale, PHQ-9 = the 9-item patient health questionnaire.

### 3.4. Regression analysis of nurses general self-efficacy and psychological resilience and mental health questionnaires

The stepwise multiple linear regression analysis shows that the regression coefficient of the resilience dimension and average monthly income is significant *(P < *.001). The toughness dimension of mental resilience and the average monthly income is the significant influencing factors of self-efficacy. (Table 4).

**Table 4:** Regression analysis of nurses self efficacy and psychological resilience and mental health questionnaires.

variable	Estimate	Se	t	*P* value	p2.5	p97.5	VIF
(Intercept)	0.927	0.081	11.386	0	0.767	1.087	
Monthly income, CNY	0.084	0.023	3.585	<.001	0.038	0.129	1.01
COVID-19 trauma							1.03
Yes	ref						
No	0.068	0.044	1.54	.124	−0.019	0.154	
Tenacity dimension	0.048	0.002	30.856	<.001	0.045	0.051	1.037

COVID-19 = corona virus disease 2019.

## 4. Discussion

The nursing profession has been under considerable strain in recent years. Austerity measures and efficiency drives have led to increased pressure on the workforce with high levels of staff shortages.^[31]^ Greater work stress led to negative emotions in nurses, high anxiety and depression, and affected their health physically and mentally.^[32]^ A sample survey for nurses across China found that 74.73% of nurses believed that their work stress was “high” or “a bit high,” and the main work stressors of nurses came from “various checks of superiors, and “low salary.”^[33]^

The professional burnout and stress of nurses in the post-pandemic era have resulted in negative consequences for organizations, hospitals, and workers.^[34]^ This study showed that 33.88% of nurses had varying degrees of depression symptoms, and 21.74% of nurses had varying degrees of anxiety symptoms 2 years after the outbreak of COVID-19. The self-efficacy of nurses was negatively correlated with anxiety and depression. Anxiety and depression hurt nurses resilience and self-efficacy. After the outbreak of COVID-19 in Wuhan in 2020, although the pandemic was quickly under control, hospitals in Wuhan were quickly put into operation. Nurses generally are not recuperated. All hospitals in Wuhan implemented strict and normalized epidemic prevention and control measures, and nurses undertook the additional work of isolation management and prevention and control of hospital infection. Nurses continued to keep working under enormous pressure to prevent COVID-19 infection. Our study also showed that 20.7% of nurses believed the COVID-19 outbreak in Wuhan caused psychological trauma. In the early days of the COVID-19 outbreak in Wuhan, a large number of patients could not go to the hospital to receive timely treatment, so the government decided that some public hospitals in Wuhan turn into the designated hospitals for COVID-19 ^[6]^, and the hospitals where we conducted our study were those designated hospitals for COVID-19 emergency cases at that time. Nurses were in close contact with COVID-19 patients for the longest time, not only undertaking the task of saving patients life and caring for their health but also their basic daily life requirements. They witnessed a large number of COVID-19 patients on the verge of death. Exposure to so many sudden traumatic events constantly for a long was a great challenge to their mental health. The impact on their psychology remained 1 year after the pandemic outbreak. According to this study, there was no significant difference in mental health among nurses in different jobs.^[35]^ This may be related to the fact that nurses had experienced strong psychological shocks when the pandemic broke out in Wuhan whether they were on the front-line or not. Two years after the outbreak of COVID-19, nurses anxiety and depression levels are lower than before, but some nurses still have psychological trauma. Studies have shown that 10% to 40% of PTSD symptoms last 1 to 3 years.^[36]^ Studies have shown that 21.3% of the general public have psychological problems in the post-epidemic era, and the psychological problems of the general public in areas with epidemics are higher than those without epidemics.^[37]^

In this study, 81.7% of nurses demonstrated moderate or high levels of self-efficacy, which is positively correlated with resilience. According to Bandura social cognitive theory, self-efficacy refers to 1’s confidence in carrying out the necessary actions to achieve desired goals.^[38]^ Self-efficacy also influences an individual’s vigilance towards potential threats; those with higher self-efficacy tend to believe they can control such threats, while those with lower self-efficacy may overestimate them.^[39]^ Resilience, on the other hand, is defined as an individual’s ability to learn and adapt in the face of adversity.^[40]^ It can mitigate the negative impact of work-related stress and prevent poor psychological health outcomes among nurses.^[41]^ During the pandemic outbreak in Wuhan, nurses demonstrated remarkable willpower, tenacity, and a strong sense of responsibility to fight the pandemic energetically and positively, which may be attributed to the support from all walks of life and government publicity at that time.^[6]^ Higher levels of self-efficacy are beneficial for disaster preparedness.^[42]^ While many research designs have noted the negative impact of the pandemic on resiliency and emotional distress,^[43,44]^ coping self-efficacy has been found to ameliorate the effects of psychological distress on nurses traumatic experiences.^[21]^ Enhancing self-efficacy may help buffer emotional exhaustion and improve communication of stress.^[45]^

The monthly income and tenacity dimension are the key factors affecting nurses self-efficacy. To be successful in their careers, nurses must possess resilience as a crucial skill that can sustain them through challenging and difficult working climates.^[46]^ Resilient individuals display intelligence and have a strong sense of self.^[47]^ The qualities associated with resilience include self-confidence, resourcefulness, curiosity, self-discipline, levelheadedness, and flexibility, together with problem-solving ability and emotional stamina.^[48]^ When the COVID-19 outbreak occurred in Wuhan, the government and hospitals adopted a series of measures to support front-line nurses in terms of material security, salary, and professional title promotion.^[49]^ These incentives can promote nurses’ self-efficacy. Two years after the outbreak of COVID-19 in Wuhan, under the normalized epidemic prevention and control measures, nurses experienced increased workload and work stress. Their income has also dropped due to the pandemic.^[50]^ Social and organizational support, including hospitals, are associated with self-efficacy.^[51,52]^In European Union countries, occupational health physicians of private and public sectors are appointed by employers to carry out evidence-based health surveillance programs to protect and prevent occupational risks, which should be normally organized within occupational health services.^[53]^Therefore, the workplace will increasingly need to implement the Total Worker Health approach.^[54]^The purpose of integrating mandatory occupational health practices and workplace health promotion programs is to reduce the high burden of work-related illnesses.^[55]^

## 5. Conclusion

This study highlights the importance of self-efficacy and resilience in promoting the psychological well-being of nurses, particularly during times of crisis such as the COVID-19 pandemic. Healthcare organizations must support their nurses and enhance their skills in coping with stress and adversity. By providing resources and support, healthcare organizations can promote the mental health and job satisfaction of their nurses, ultimately leading to improved patient outcomes and quality of care. Twos year after the outbreak of COVID-19 in Wuhan, anxiety and depression among nurses were lower than during the initial outbreak period, and most nurses demonstrated moderate or high levels of self-efficacy. However, hospital management should continue to pay attention to the psychological state of nurses, the trauma they have experienced, and their decreased income due to the pandemic outbreak, and take various measures to help improve their mental health. It is important to note that this study is limited by its single-center design, and the conclusions should be interpreted with caution.

Overall, the findings of this study suggest that healthcare organizations should prioritize the development of self-efficacy and resilience among their nurses. By doing so, they can not only improve the well-being of their nurses but also enhance patient outcomes and quality of care.

## Author contributions

**Conceptualization:** Yan Yun Xianyu.

**Data curation:** Ying Zhi Wan.

**Formal analysis:** Ying Zhi Wan.

**Writing – original draft:** Wen Li.

**Writing – review & editing:** Wen Li.
